# Induction of E6AP by microRNA-302c dysregulation inhibits TGF-β-dependent fibrogenesis in hepatic stellate cells

**DOI:** 10.1038/s41598-019-57322-w

**Published:** 2020-01-16

**Authors:** Ji Young Kim, Kyu Min Kim, Ji Hye Yang, Sam Seok Cho, Seung Jung Kim, Su Jung Park, Sang‐Gun Ahn, Gum Hwa Lee, Jin Won Yang, Sung Chul Lim, Keon Wook Kang, Sung Hwan Ki

**Affiliations:** 1 College of Pharmacy, Chosun University, Gwangju, 61452 Republic of Korea; 2 College of Korean Medicine, Dongshin University, Naju, Jeollanam-do 58245 Republic of Korea; 3 Department of Pathology, College of Dentistry, Chosun University, Gwangju, 61452 Republic of Korea; 4 College of Pharmacy, Woosuk University, Wanju, Jeonbuk 55338 Republic of Korea; 5 College of Medicine, Chosun University, Gwangju, 61452 Republic of Korea; 6 College of Pharmacy and Research Institute of Pharmaceutical Sciences, Seoul National University, Seoul, 08826 Republic of Korea

**Keywords:** Hepatic stellate cells, Liver fibrosis

## Abstract

Hepatic stellate cells (HSCs) are essential for liver fibrosis. E6 associated protein (E6AP) is one of the E3-ubiquitin-protein ligase and has been studied in proliferation and cellular stress. Currently, no information is available on the role of E6AP on transforming growth factor-β (TGF-β) signaling and hepatic fibrogenesis. This study examined whether E6AP is overexpressed in activated HSCs, and if so, its effect on hepatic fibrogenesis and the molecular mechanism. E6AP was expressed higher in HSCs than hepatocytes, and was up-regulated in activated HSCs, HSCs from the livers of carbon tetrachloride-injected mice, or TGF-β-treated LX-2 cells. The TGF-β-mediated E6AP up-regulation was not due to altered mRNA level nor protein stability. Thus, we performed microRNA (miRNA, miR) analysis and found that miR-302c was dysregulated in TGF-β-treated LX-2 cells or activated primary HSCs. We revealed that miR-302c was a modulator of E6AP. E6AP overexpression inhibited TGF-β-induced expression of plasminogen activator inhibitor-1 in LX-2 cells, albeit it was independent of Smad pathway. Additionally, E6AP inhibited TGF-β-mediated phosphorylation of mitogen-activated protein kinases. To conclude, E6AP overexpression due to decreased miR-302c in HSCs attenuated hepatic fibrogenesis through inhibition of the TGF-β-induced mitogen-activated protein kinase signaling pathway, implying that E6AP and other molecules may contribute to protection against liver fibrosis.

## Introduction

Liver fibrosis, derived from a variety of etiologies, such as hepatitis B or C virus infection, chronic alcohol abuse, non-alcoholic steatohepatitis, cholestasis, and autoimmune hepatitis, can advance to fibrosis and cirrhosis, which are major causes of morbidity and mortality worldwide^1^. Upon repeated injury, the liver undergoes a wound-healing response, leading to the accumulation of excessive deposition of extracellular matrix (ECM) and impaired organ function^2^.

During this process, hepatic stellate cells (HSCs) trans-differentiate from quiescent cells with vitamin A, to highly proliferative myofibroblastic cells, and these activated cells are crucial sources for fiber accumulation and contribute to liver fibrosis^3^. Thus, decrease of the number of activated HSCs is an attractive application for anti-fibrotic therapy^4^. Unfortunately, to date, there is no established way to modulate HSC activation. Hence, it is necessary to identify new regulatory targets and the underlying mechanisms of this process to treat liver fibrosis.

Transforming growth factor-β (TGF-β) is the most potent profibrogenic cytokine for activated HSCs^5^. TGF-β triggers phenotypical HSC transdifferentiation by paracrine and autocrine action, and directly induces collagen I (Col I) expression and α-smooth muscle actin (α-SMA) stress fiber organization^6^. TGF-β signals through transmembrane receptors, consisting of type I and II heterodimers, to initiate downstream signaling via Smad proteins and Smad-independent proteins such as mitogen activated protein kinase (MAPK) [extracellular signal-regulated kinase (ERK), c-jun N-terminal kinase (JNK) and p38]^7–9^. These pathways affect the expression of TGF-β-dependent target genes, encoding ECM components^10^.

The ubiquitin-proteasome pathway has emerged as a critical post-translational modification mechanism that regulates cell proliferation and differentiation, signal transduction, and apoptosis^11^. Protein degradation via ubiquitin proteasome system is delicately controlled within the cell to maintain protein homeostasis and eliminate misfolded or damaged proteins, and involves a cascade of enzymes called E1 (ubiquitin-activating enzyme), E2 (ubiquitin-conjugating enzyme) and E3 (ubiquitin-ligase)^12^. The effects of ubiquitination-triggered degradation are mainly achieved by E3 ubiquitin ligases (E3 ligase), which are crucial for the selective recognition of target proteins and subsequent protein degradation. Additionally, several E3 ligases (e.g., Smad ubiquitin regulatory factor 2 (Smurf 2)^13^, S-phase kinase-associated protein 2 (SKP-2), and synoviolin^14^) play an important role in liver fibrosis. Because the ubiquitin-proteasomal degradation pathway tightly regulates TGF-β signaling^15–17^, the mainly involved in HSC activation and ECM accumulation, E3 ligase may have profound effects on the progression of liver fibrosis.

E6-associated protein (E6AP), a member of the HECT (homologous with E6AP C-terminus) family protein, is known as an E3 ligase involved in the degradation of p53 and various other cell-cycle regulatory proteins^18^. E6AP is encoded by the *Ube3A* locus, which is mutated in a neurological disorder called Angelman Syndrome^19^. A number of studies have demonstrated that E6AP affects the malignant potential of cancer cells via controlling cell proliferation, senescence and cellular response to oxidative stress^20–22^. Although E6AP has been known to exacerbate liver cancer by promoting hepatocellular proliferation^23^, little information is available on the role of E6AP in liver pathophysiology. Especially, the involvement of E6AP and its mechanism in the regulation of TGF-β signaling and fibrogenesis in HSCs has not been studied.

In this study, we investigated whether TGF-β signaling upregulates E6AP expression in HSCs, and if so, what the subsequent impact is on HSC activation and how it is regulated. We found that E6AP abundantly expressed in HSCs compared to hepatocytes, and was induced in activated HSCs due to dysregulation of a specific microRNA (miRNAs, miR), which suppressed liver fibrogenesis. Ectopic expression of E6AP inhibited TGF-β-mediated activation of MAPKs, but not Smad phosphorylation. In addition, we showed that c-Jun or c-Fos-dependent AP-1 activity is related to the anti-fibrogenic effect of E6AP. Our findings provide a novel role for E6AP in HSC activation and extends the basic scientific information on liver fibrosis.

## Results

### E6AP was up-regulated in HSCs and fibrotic liver

**Figure 1:**
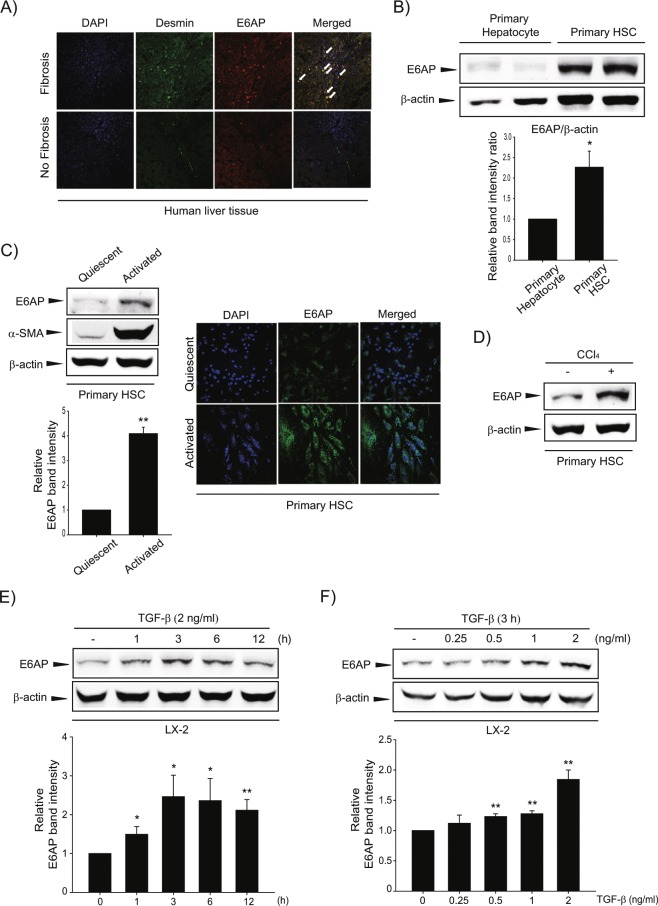
Upregulation of E6AP during HSC activation. (**A**) Immunostaining of E6AP and desmin (magnification: 40×). White arrows indicate colocalization of E6AP and desmin. (**B**) E6AP expression in mouse primary hepatocyte and quiescent hepatic stellate cells (HSCs). E6AP and β-actin levels were assessed by scanning densitometry. The data represents the mean ± standard error (SE) (*n* = 3, significant different versus primary hepatocyte, *p < 0.05). (**C**) left: E6AP expression in quiescent or activated primary HSCs. Mouse primary HSCs were extracted and cultured in medium for 0 (quiescent) or 7 (activated) days. Immunoblotting was done on the cell lysates (20 μg each). α-smooth muscle actin (α-SMA) was detected to confirm HSC activation and β-actin was verified for equal loading of proteins. At least three separate samples were used for experiments. The data represents the mean ± SE (*n* = 3, significant different versus quiescent, **p < 0.01). Right: Immunostaining for E6AP. Samples were prepared as described in (**C**), left. (**D**) E6AP level in primary HSCs obtained from CCl_4_-treated mice. Mice were injected with 0.5 mg/kg CCl_4_ for 24 h (*n* = 3). Immunoblot of E6AP was performed. (**E**) The effect of transforming growth factor-β (TGF-β) on E6AP upregulation. The time courses of E6AP expression in TGF-β (2 ng/mL)-exposed LX-2 cells. The data represents the mean ± SE (*n* = 3, significant different versus 0 h, *p < 0.05 and **p < 0.01). (**F**) The effect of various concentrations of TGF-β on E6AP induction in LX-2 cells. E6AP protein in the lysates of cells, incubated with 0.25-2 ng/mL TGF-β for 3 h, was immunoblotted. The data represents the mean ± SE (*n* = 3, significant different in comparison with control, **p < 0.01).

We first examined E6AP and desmin, a marker of HSC activation in the cirrhotic and adjacent normal tissue samples from patients with cancer to find the biological significance of E6AP in a clinical situation. Expression of E6AP and desmin were higher in the cirrhotic samples and were seen in similar regions of the specimens (Fig. 1A). We compared E6AP expression in different types of hepatic cells. We found that E6AP showed higher expression, in HSCs than in hepatocytes (Fig. 1B and Supplemntary Fig. 1). Additionally, E6AP was up-regulated in primary HSCs during culture activation with the increase of α-SMA, an HSC trans-differentiation marker (Fig. 1C, left). Consistently, primary activated HSCs showed a significant increase in immunostaining of E6AP compared to quiescent HSCs (Fig. 1C, right). Furthermore, we isolated HSCs from mice treated with vehicle or carbon tetrachloride (CCl_4_). E6AP was up-regulated in HSCs from CCl_4_-injected mice (Fig. 1D). Next, we investigated E6AP expression after TGF-β stimulation, for different time periods and varying concentrations, in LX-2 cells, immortalized human HSC cell lines. E6AP was found to increase after 1–12 h of TGF-β treatment and peaked at 3 h (Fig. 1E). Additionally, we observed that E6AP was markedly induced by TGF-β treatment and reached a maximum at 2 ng/mL of TGF-β (Fig. 1F). These results suggest that E6AP is overexpressed in activated HSCs during liver fibrogenesis.

### E6AP induction was not controlled by transcriptional regulation or protein stability

**Figure 2:**
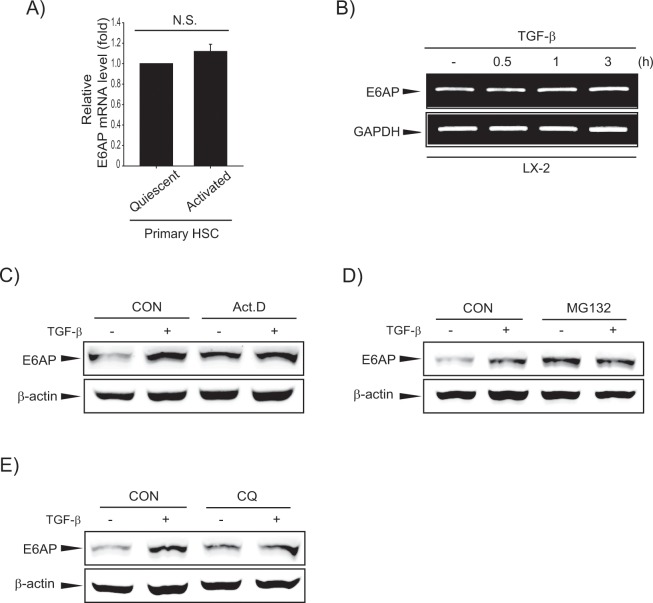
E6AP induction by neither transcriptional mechanism nor protein stability. (**A**) Real-time PCR analysis. The E6AP transcript was measured, with GAPDH used as a housekeeping gene in quiescent or activated primary HSCs. The data represents the mean ± SE (*n = *3, N.S., not significant). (**B**) RT-PCR analysis. TGF-β (2 ng/mL) was exposed to LX-2 cells for 0.5-3 h. Transcriptional levels of E6AP were determined by RT-PCR using GAPDH as an internal control. (**C**) The effect of actinomycin-D (Act.D) on E6AP induced by TGF-β in LX-2 cells. The cells were incubated with Act.D (5 μg/mL) in the presence or absence of TGF-β. The E6AP level was analyzed after TGF-β (2 ng/mL) treatment for 6 h. (**D**) The effect of proteasomal inhibition on E6AP induced by TGF-β. LX-2 cells were pretreated with of MG132 (10 ng/mL) for 0.5 h, and then were incubated with TGF-β (2 ng/mL) for 3 h, and then E6AP level was detected by immunoblotting. (**E**) The effect of lysosomal inhibition on E6AP induced by TGF-β. LX-2 cells were pretreated with chloroquine (CQ, 100 μg/mL) for 1 h, and then were treated with TGF-β (2 ng/mL) for 3 h, and then E6AP expression was evaluated by immunoblotting.

To delineate the molecular mechanism governing E6AP overexpression, we investigated E6AP mRNA levels, and discovered that they were not changed in activated HSCs or TGF-β-treated LX-2 cells (Fig. 2A,B). Furthermore, we treated LX-2 cells with a transcription inhibitor, actinomycin-D (Act.D) alone or in combination with TGF-β, and further demonstrated that altered E6AP expression was not due to transcriptional regulation (Fig. 2C and Supplemntary Fig. 2). On performing additional assays using a proteasomal inhibitor (MG132, MG) and a lysosomal inhibitor (chloroquine, CQ), we found that E6AP induction by TGF-β was not affected (Fig. 2D,E). These results imply that E6AP expression was not controlled by transcriptional regulation or protein stability.

### MicroRNA-302c is identified as a novel regulator of E6AP

**Figure 3:**
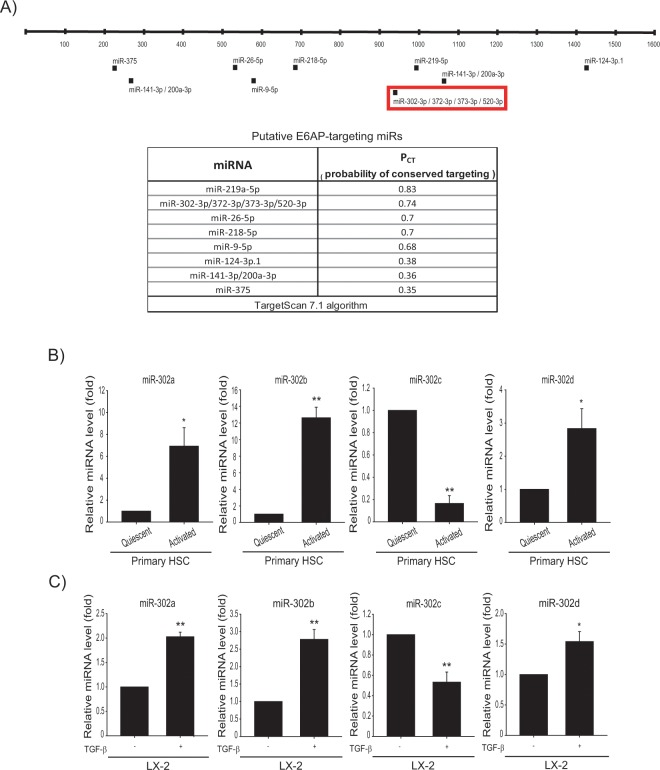
Decreased miR-302c level upon TGF-β treatment. (**A**) The locations of the predicted miRNA binding sites within the 3′-UTR of E6AP mRNA. (**B**) Real-time RT-PCR assays for the miR-302a/b/c/d-3p in quiescent or activated primary HSCs. Data were normalized against the levels of U6 small RNA. The data represents the mean ± SE (*n* = 3, significant as compared with primary quiescent HSCs, *p < 0.05 and **p < 0.01). (**C**) Real-time RT-PCR assays for the candidate miRs in LX-2 cells. LX-2 cells were incubated with TGF-β (2 ng/mL) for 30 min. The data represents the mean ± SE (*n* = 3, significant whe*n* in comparison with vehicle-treated LX-2 cells, *p < 0.05 and **p < 0.01).

**Figure 4:**
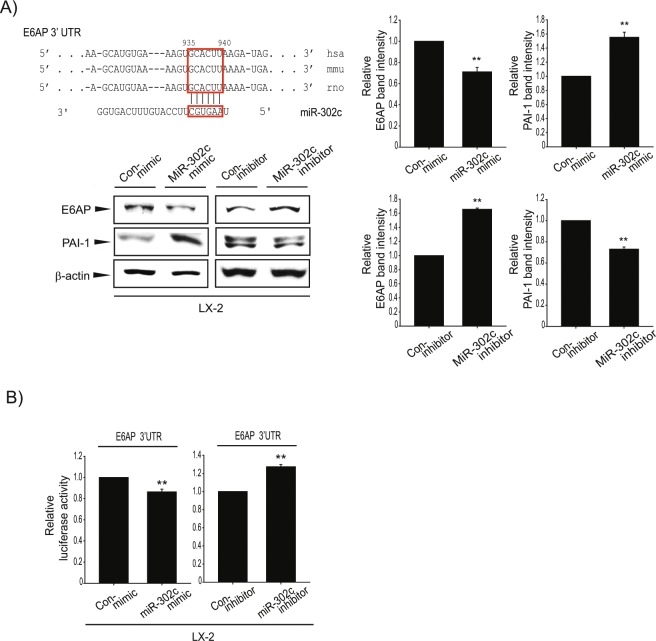
Inhibition of E6AP translation by miR-302c. (**A**) Upper: Alignments of miR-302c binding to the 3′-UTRs of E6AP mRNAs. Lower: Effect of miR-302c on E6AP and expression of fibrogenic gene. LX-2 cells were transfected with control mimic (Con-mimic) or miR-302c mimic for 24 h and transfected with control inhibitor (Con-inhibitor) or miR-302c inhibitor for 24 h. E6AP expression and PAI-1 protein level in the lysates of the LX-2 cells, transfected with miR-302c mimic or inhibitor (and their respective controls) was detected by immunoblotting. For A (right), E6AP and PAI-1 levels were assessed by scanning densitometry. The data represents the mean ± SE (*n* = 3-4, significant when compared with respective controls, **p < 0.01). (**B**) Effect of miR-302c mimic or inhibitor on E6AP 3′-UTR reporter assays. Left: Cells were transfected with Con-mimic or miR-302c mimic, and E6AP 3′-UTR reporter. Right: Cells were transfected with Con-inhibitor or miR-302c inhibitor, and E6AP 3′-UTR reporter. Results represent the mean ± SE (*n* = 3, sig*n*ificant when compared with miR-302c mimic or inhibitor-transfected cells, **p < 0.01).

miRNAs are small non-coding RNA molecules (containing about 22 nucleotides) that play important gene regulatory roles via base-pairing with complementary sequences to the mRNAs of protein-coding genes to direct mRNA silencing. Inhibitory modulation of the target genes leads to decrease in translation efficiency and/or reduce mRNA level by binding to the 3′-untranslated region (UTR) of the target mRNA^24,25^. We searched for putative miRNAs targeting 3′-UTR of E6AP using the TargetScan 7.1 algorithm (http://www.targetscan.org); miR-219-5p, miR-302-3p/372-3p/373-3p/520-3p, miR-26-5p, miR-218-5p, miR-9-5p, miR-124-3p, miR-141-3p/200a-3p, miR-375 (Fig. 3A) emerged as the candidate miRNAs regulating the expression of E6AP. Of these, miR-219-5p was predicted as a miRNA with highest affinity to E6AP according to the P_CT_. The level of miR-219-5p was decreased in TGF-β-treated LX-2 cells as well as activated primary HSCs compared to respective controls. However, we found that miR-219-5p did not affect E6AP expression after miR-219-5p modulation (data not shown). Hence, we focused on miR-302-3p/372-3p/373-3p/520-3p, the miRNAs with the second highest probability for matching E6AP. Of these miRNA candidates, miR-302c was significantly decreased in activated primary HSCs (Fig. 3B). Moreover, TGF-β treatment of LX-2 cells substantially decreased the level of miR-302c (Fig. 3C). Other members of the miR-302 family were not decreased in activated primary HSCs. Additionally, we found that highly conserved miR-302c recognition sites were present in the 3′-UTR region of E6AP mRNA (Fig. 4A, upper). To demonstrate whether miR-302c directly inhibits E6AP synthesis, E6AP expression was examined after miR-302c modulation, using its mimic or inhibitor. E6AP levels were reduced by miR-302c mimic transfection and conversely increased by miR-302c inhibitor transfection in LX-2 cells (Fig. 4A, lower and Supplemntary Fig. 3). We then assessed the functional role of miR-302c in fibrogenesis; miR-302c modulation changed plasminogen activator inhibitor 1 (PAI-1) expression levels. Additionally, miR-302c inhibitor or mimic transfection resulted in appropriate change in luciferase activity from the E6AP-3′-UTR in LX-2 cells (Fig. 4B). These results indicated that E6AP overexpression was derived from miR-302c dysregulation in activated HSCs.

### E6AP overexpression inhibits TGF-β-induced hepatic fibrogenesis

**Figure 5:**
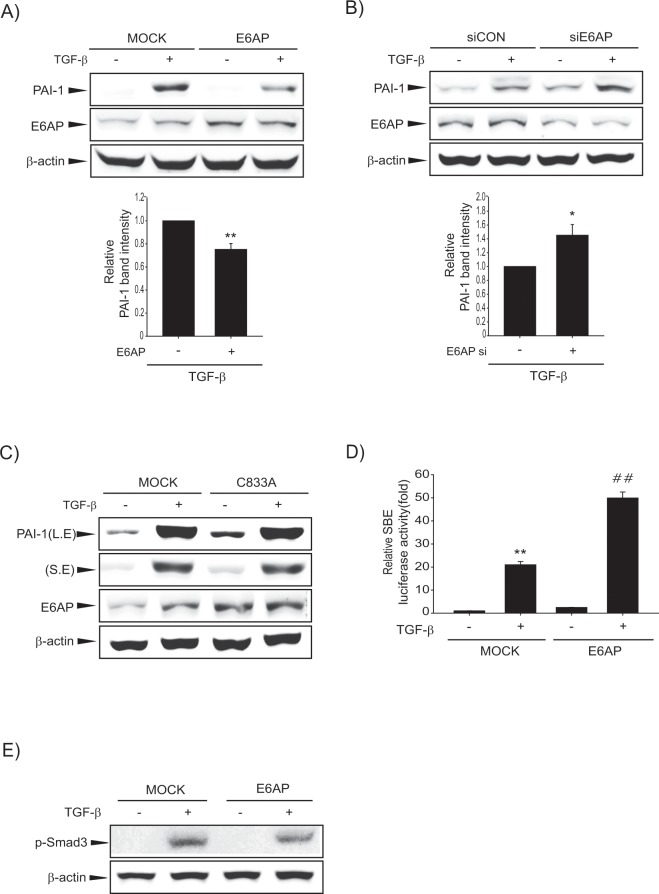
The effect of E6AP on hepatic fibrogenesis in LX-2 cells. (**A**) Effect of E6AP on TGF-β-mediated fibrogenic gene expression. Cells were transfected with pCMV4 (MOCK) or pCMV4-E6AP (E6AP) for 24 h and then exposed to TGF-β (1 ng/mL) for 3 h. PAI-1 was assessed by immunoblotting. Overexpression of E6AP was confirmed by immunoblotting. PAI-1 levels were assessed by scanning densitometry. The data represents the mean ± SE (*n* = 3, significant different versus MOCK, **p < 0.01). (**B**) Effect of E6AP knockdown on TGF-β-related fibrogenic gene expression. Cells were transfected with control siRNA (siCon) or E6AP siRNA (siE6AP) for 24 h and then treated with TGF-β (1 ng/mL) for 3 h. The data represents the mean ± SE (*n* = 3, significant different versus siCON, *p < 0.05). (**C**) Effect of E6AP mutant (C833A) on TGF-β-induced fibrogenic gene expression. Cells were transfected with MOCK or C833A for 24 h and then incubated with TGF-β (1 ng/mL) for 3 h. Immunoblotting was done to detect PAI-1 level. (**D**) Effect of E6AP on Smad binding element (SBE) luciferase activity by TGF-β treatment. LX-2 cells were transfected with SBE luciferase construct. Then, cells were treated with TGF-β (1 ng/mL) for 3 h. The data represents the mean ± SE (*n* = 3, significant whe*n* in comparison with vehicle-treated controls, **p < 0.01; significant when compared with TGF-β alone, ^##^*p* < 0.01). (**E**) Effect of TGF-β-induced Smad3 phosphorylation by E6AP. Cells were transfected as described above and exposed to TGF-β (1 ng/mL) for 30 min, and the lysates were immunoblotted for detection of Smad3 phosphorylation. At least triple replicates were preformed to verify the results.

Next, we explored the role of E6AP on TGF-β-induced fibrogenic gene expression and related signaling pathways. TGF-β treatment of MOCK-transfected LX-2 cells increased PAI-1 expression, which was attenuated by E6AP overexpression (Fig. 5A and Supplemntary Fig. 4). Similarly, TGF-β-induced PAI-1 expression was enhanced by E6AP siRNA in LX-2 cells compared to control siRNA-transfected LX-2 cells (Fig. 5B). An additional assay was performed with a catalytically inactive mutant of E6AP (termed C833A), which cannot form a thiol ester with ubiquitin^26^. Interestingly, C833A exerted no effect on PAI-1 expression against E6AP (Fig. 5C). To determine the possible link between E6AP and TGF-β signaling, we checked for luciferase activity from a Smad-binding element (SBE)-driven reporter. E6AP failed to repress SBE luciferase activity by TGF-β treatment (Fig. 5D). Further, when we investigated the effect of E6AP on TGF-β-dependent Smad phosphorylation, we did not observe any effect on Smad3 phosphorylation against E6AP (Fig. 5E). Hence, these data imply that E6AP attenuated TGF-β-dependent fibrogenesis via Smad-independent pathway.

### AP-1 inhibition by E6AP contributes to antifibrogenic effect

**Figure 6:**
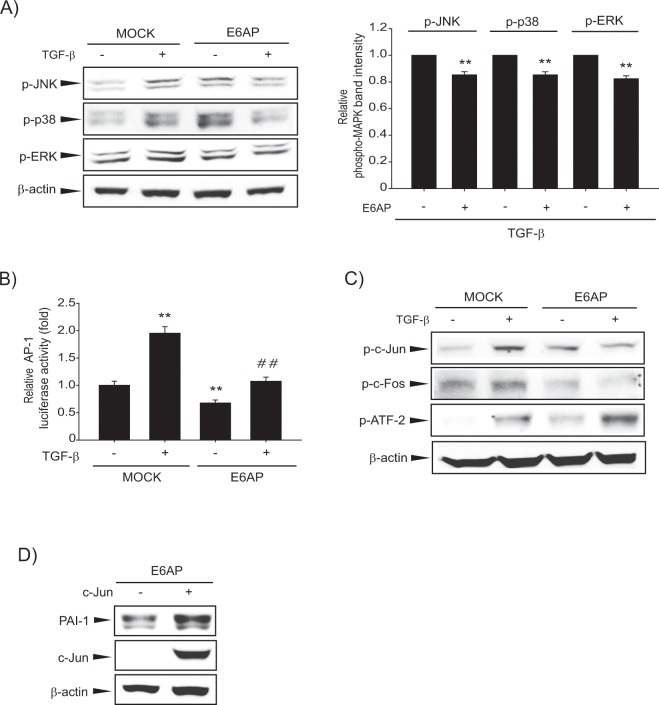
Association of AP-1 with the effect of E6AP on hepatic fibrogenesis in LX-2 cells. (**A**) Effect of E6AP on TGF-β-induced mitogen-activated protein kinase (MAPK) phosphorylation. LX-2 cells were transiently transfected with MOCK or E6AP for 24 h and then exposed to TGF-β (1 ng/mL) for 3 min. Phospho-MAPK levels were assessed by scanning densitometry. The data represents the mean ± SE (*n = *3, significant when in comparison with respective TGF-β alone, **p < 0.01). (**B**) Effect of E6AP on AP-1 luciferase activity by TGF-β incubation. LX-2 cells were transfected with AP-1 luciferase plasmid in combination with E6AP (or MOCK) and incubated with TGF-β (1 ng/mL) for 3 h. Results represent the mean ± SE (*n* = 3, significant when in comparison with vehicle-treated controls, **p < 0.01; significant when compared with TGF-β alone, ^##^*p* < 0.01). (**C**) Effect of E6AP on TGF-β-mediated phosphorylation of AP-1 transcription factors. Cells were transfected as described above and treated with 2 ng/mL TGF-β for 5 min, and the c-Jun phosphorylation was detected by immunoblotting. (**D**) The role of AP-1 transcription factors in E6AP-mediated antifibrogenic effect. Cells were transfected with E6AP in combination with c-Jun (or MOCK). Ectopic expression of c-Jun was confimed by immunoblotting with lysates from transfected cells. Results were confirmed by repeating the experiments at least triple replicates.

To reveal the other signaling pathway that controls the antifibrogenic effect of E6AP, we sought to determine the role of mitogen-activated protein kinase (MAPK) signaling since TGF-β might activate other molecules such as MAPKs^27^. The treatment of MOCK-transfected LX-2 cells with TGF-β increased MAPK phosphorylation including JNK, p38, and ERK. However, E6AP overexpression suppressed the increased MAPK activity by TGF-β (Fig. 6A and Supplemntary Fig. 5). Next, we measured AP-1-dependent luciferase activity to assess the inhibitory effect of E6AP on TGF-β-mediated AP-1 activation. We found that increased AP-1 luciferase activity by TGF-β was suppressed by E6AP overexpression (Fig. 6B). AP-1 is a transcription factor composed of the Jun and Fos or activating transcription factor (ATF), and acts as a heterodimer of Jun and Fos^28^. Interestingly, E6AP overexpression attenuated the effect of TGF-β on phosphorylation of c-Jun and c-Fos, but not ATF2 (Fig. 6C). Overexpression of c-Jun reversed the antifibrogenic effect of E6AP, corroborative of the connections between c-Jun and E6AP (Fig. 6D). Our results demonstrated that E6AP-mediated antifibrogenesis in HSCs might be associated with attenuation of c-Jun-dependent AP-1 activation.

## Discussion

TGF-β is one of the most well-studied profibrotic cytokines that promotes the synthesis and deposition of diverse ECM components such as a variety of collagen, laminin, and fibronectin during hepatic fibrogenesis^10^. E3 ligases can interact with various substrates which are associated with TGF-β signaling and regulate their expression levels^29^. Although E6AP has been reported to regulate important cellular processes, including signal transduction, transcription, cell survival, apoptosis, and DNA repair^30^, its expression as well as its role in liver fibrosis remains to be elucidated. In the current study, we discovered for the first time, that E6AP is elevated during HSC activation, suggesting a close relationship between E6AP and liver fibrosis. Data demonstrating E6AP upregulation in HSCs isolated from the CCl_4_-treated mice and TGF-β-treated LX-2 cells, also strengthened our findings. Our results show that E6AP expression was more abundant in HSCs than in hepatocytes, implying an important role of E6AP in HSC biology.

MiRNAs have recently emerged as a class of small RNAs that negatively regulate gene expression by binding to the 3′-UTR of their target mRNAs. They regulate normal developmental processes, including differentiation and proliferation, as well as hepatic disease development and progression with multiple and diverse targets^31^. In addition, other aspects of HSC biology are also governed by miRNAs; miR-27a/b regulates HSC transactivation, miR-15b/16 are involved in HSC proliferation, and the miR-29 family inhibits a number of fibrosis-related proteins^32–34^. Our study identified miR-302c as a new regulator of E6AP that controls TGF-β-mediated fibrogenesis during HSC activation. We observed that E6AP induction was not due to either transcriptional regulation or protein stability. These outcomes encouraged us to investigate the involvement of miRNAs, another major post-transcriptional mechanism. miR-302c was markedly downregulated during HSC activation and TGF-β treatment in HSCs, and that miR-302c suppressed E6AP expression via binding to 3′-UTR of E6AP mRNA, supports our conclusion that miR-302c dysregulation is a regulatory mechanism of E6AP overexpression in HSCs. The effect of miR-302c on fibrogenic genes indicated that miR-302c dysregulation affected fibrogenesis in HSCs through E6AP regulation. Despite the known effect of miR-302c on self-renewal and pluripotency processes, neural differentiation, and progression of liver cancer^35^, the role of miR-302c in the HSC activation has never been studied. Therefore, our finding, demonstrating a role of miR-302c in the regulation of E6AP expression, may provide a novel strategy for antifibrotic therapy.

E6AP, which is known as a HECT-type E3 ligase, has a variety of substrate proteins: p53^18^, β-catenin^36^, ErBb2^37^, peroxiredoxin 1^38^, CCAAT/enhancer binding protein α, and TSC2^39^. Our finding, that a catalytically inactive mutant of E6AP is unable to suppress TGF-β-mediated PAI-1 induction, suggests that E6AP ubiquitinates TGF-β signaling-related proteins. In an effort to determine a direct target of E6AP, several components of TGF-β signaling (Smad 2/3, Smad7, TGF-β receptor I/II) were examined after E6AP overexpression, with or without TGF-β treatment. Unfortunately, we observed that none of the above mentioned proteins were affected by E6AP modulation (data not shown). Therefore, further research is needed to identify the substrate of E6AP, during HSC activation.

Specific serine/threonine kinase receptors and Smads are well-established effectors in TGF-β signaling, but it is also accepted that additional TGF-β responses control the activation of Smad2/3-independent pathways such as MAPK^9,40^. Our data indicate that inhibition of TGF-β-mediated HSC activation by E6AP rely on antagonizing effect of MAPK activation. Unexpectedly, E6AP rather increased SBE luciferase activity. These results should be clarified in further study. JNK, p38, and ERK activation induces HSC proliferation and HSC activation in response to TGF-β^41-43^. However, former studies have demonstrated that JNK and p38 have distinct effects on HSC biology^44–46^. Despite reports on the controversal roles of MAPKs in HSCs^44–46^, our findings indicate that antifibrogenic effect of E6AP were related to attenuation of phosphorylation of JNK, p38, or ERK.

AP-1 family is a dimeric transcription factors composed of Jun, Fos or ATF^47,48^. Specific AP-1 components may regulate different target genes and thus execute distinct biological functions. In addition to regulation by heterodimerization between Jun, Fos and ATF proteins, AP-1 activity was regulated through interactions with specific protein kinases and a variety of transcriptional coactivators^49^. Hence, we suppose the suppressed Jun or Fos activation but not ATF phosphorylation, may be derived from the direct and/or indirect effects of multiple kinases on E6AP-dependent HSC activation. Indeed, some of AP-1 components were affected by altered activities of MAPKs in several studies by us and others^50–52^. Hence, we suppose the result of Fig. 6C, which E6AP specifically inhibited Jun or Fos activation but not ATF, may be derived from the direct and/or indirect effects of multiple kinases on E6AP-dependent HSC inhibition.

**Figure 7:**
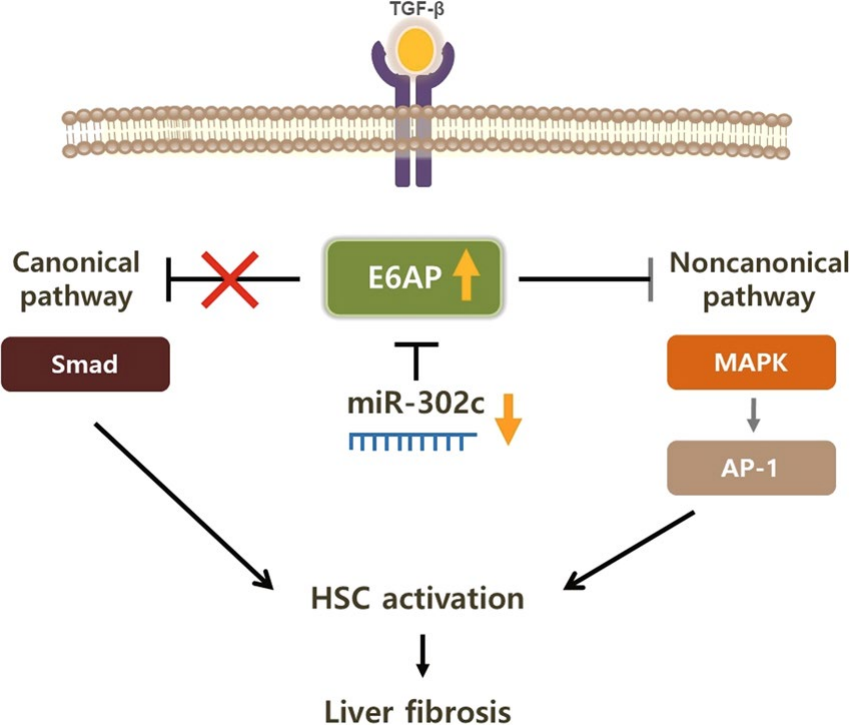
A schematic diagram illustrating the proposed anti-fibrotic mechanism by which E6AP induction by miR-302c dysregulation.

Collectively, our study reveals that E6AP is overexpressed during trans-differentiation of HSCs, mediated by miR-302c dysregulation, and this event suppresses MAPK phosphorylation and subsequent c-Jun-dependent AP-1 activation (Fig. 7). These findings provide new insight into the regulation of a key signaling pathway, which attenuates hepatic fibrogenesis and consequent liver fibrosis.

## Materials and Methods

### Materials

Antibody against E6AP was obtained from Santa Cruz Biotechnology (SantaCruz, CA, USA). PAI-1 antibody was purchased from BD Bioscience (San Jose, CA, USA). Phospho-JNK1/2, phospho-ERK, phospho-p38, phospho-Smad3, phospho-c-Jun, and phospho-c-Fos antibodies were procured from Cell Signaling Technology (Danvers, MA, USA). Horseradish peroxidase-conjugated goat anti-mouse and anti-rabbit antibodies were purchased from Invitrogen (Carlsbad, CA, USA). α-SMA, and β-actin antibodies, Z-Leu-Leu-Leu-al (MG132), chloroquine, and actinomycin-D were obtained from Sigma (St. Louis, MO, USA). TGF-β was provided from R&D Systems (Minneapolis, MN, USA).

### Patient samples

Human liver samples were obtained from 10 patients who had been diagnosed with cancer, with or without cirrhosis, by histologic examination and ultrasonography in Chosun University Hospital in South Korea, as used in the previous study^53^. The study protocol was approved by the institutional review board of Chosun Medical Center (#2013-04-005). Informed consent was obtained from all subjects, and all methods were carried out in accordance with the relevant guidelines and regulations of Chosun Medical Center Ethics Committee.

### Cell culture

The LX-2 cell (an immortalized human HSC line) were generously supplied from Dr. S.L. Friedmann (Mount Sinai School of Medicine, NY, USA). The cells were grown in a medium containing DMEM, 10% fetal bovine serum (FBS; Atlas Biologicals, Fort Collins, CO, USA), 5% penicillin-streptomycin at 37 °C, in an humidified atmosphere with 5% CO_2_. Cells were then washed twice with ice-cold phosphate buffered saline, before sample preparation.

### Isolation of Hepatocytes and HSCs

Hepatocytes and HSCs were isolated from the liver of 8 week old mice (Oriental Bio, Sungnam, South Korea) as previously reported^54,55^. The protocols of all animal experiments were reviewed and approved by the Animal Care and Use Committee of Chosun University (CIACIC 2018-S0048). After intubation in the portal vein, the livers were perfused *in situ* with Ca^2+^-free Hank’s balanced saline solution at 37 °C for 15 min and then perfused with solution containing 0.05% collagenase and Ca^2+^ for 15 min, at a flow rate of 10 mL/min. The perfused livers were minced, filtered through 70 μm cell strainer (BD Bioscience), and centrifuged at 50* g* for 3 min to separate the hepatocytes. Hepatocytes were resuspended in DMEM, supplemented with 10% FBS, 100 U/mL penicillin and 100 μg/mL streptomycin, 5 mM HEPES, and 10 nM dexamethasone. HSCs were isolated according to a previously published method^56^. Briefly, the supernatant was further centrifuged at 500 *g* for 10 min, resuspended in Ficoll plus Percoll (1:10, GE Healthcare, Chicago, IL, USA), and centrifuged at 1,400 *g* for 17 min. HSCs were collected from the interface. Quiescent HSCs were cultured for 0 day, and activated HSCs were cultured for 7 days.

### Immunoblot analysis

Total cell lysates were prepared as previously reported^51^. Briefly, the cell lysates were centrifuged at 3,000 g for 3 min and allowed to lysis after the addition of lysis buffer. Lysates were centrifuged at 10,000 g for 10 min to obtain supernatant and were at −70 °C. Protein samples were separated by electrophoresis and transferred to a nitrocellulose membrane. The nitrocellulose membrane was blocked using 5% non-fat dried milk in Tris-buffer saline and Tween 20 (TBST) (20 mM Tris-HCl, 150 mM NaCl, and 0.1% Tween 20, pH 7.5) for 1 h and exposed with the indicated primary antibody overnight at 4 °C. After washing with TBST buffer, membranes were incubated with a horseradish peroxidase-conjugated secondary antibody (Invitrogen, San Diego, CA, USA). Protein bnads were visualized using enhanced chemiluminescence detection kit (Amersham Biosciences, Buckinghamshire, UK) using LAS 4000 mini (GE Healthcare). Equal protein loading was verified using β-actin. Scanning densitometry was done with an Image J (National Institutes of Health, Bethesda, MD, USA).

### RNA isolation, semicomparative RT-PCR, and quantitative RT-PCR analysis

Total RNA was extracted with TRIzol (Invitrogen), and the RNA (2 μg each) was reverse-transcribed to obtain cDNA using an oligo-d(T)_18_ primers and a cDNA synthesis kit (Bioneer, Daejeon, Korea) with a thermal cycler (Bio-Rad, Hercules, CA, USA). Amplified cDNAs were separated, performed ethidium bromide staining, and visualized in gel documentation system. Quantitative PCR was done using StepOne system (Applied Biosystems, Foster City, CA, USA) and SYBR green premix (Applied Biosystems). The relative levels of reverse-transcribed mRNAs were determined based on the threshold cycle value. A melting curve analysis of each amplicon was done to verify its accuracy. Primer sequences were as follows: human E6AP sense 5′-AGGCCATCACGTATGCCAAA-3′, and antisense 5′-AGGGAGGCACAGACATAGGT-3′; mouse E6AP sense 5′-ACTGGGGAAAGTGCATCTGG-3′, and antisense 5′-TGCTGCAACACTGATCGAGT-3′; human GAPDH sense 5′-GAAGGTGAAGGTCGGAGTC-3′, and antisense 5′-GAAGATGGTGATGGGATTTC-3′; mouse GAPDH sense 5′-TGCCCCCATGTTTGTGATG-3′, and antisense 5′-TGTGGTCATGAGCCCTTCC-3′. The mRNA expression data were normalized on those of GAPDH. Quantitative RT-PCR for miRNA was done using miScript SYBR Green PCR kit (Qiagen, Hiden, Germany) according to the manufacturer’s instruction. All results were normalized to U6 small RNA levels measured using the Hs_RNU6B_2 miScript Primer Assay kit (Qiagen). Primer sequences were as follows: miR-302a-3p, 5′-TAAGTGCTTCCATGTTTTGGTGA-3′; miR-302b-3p, 5′-TAAGTGCTTCCATGTTTTAGTAG-3′; miR-302c-3p, 5′-TAAGTGCTTCCATGTTTCAGTGG-3′; miR-302d-3p, 5′-TAAGTGCTTCCATGTTTGAGTGT-3′. The relative expression values were normalized to the internal control using 2^−△△Ct^.

### Transient transfection and siRNA knockdown experiment

Plasmids encoding E6AP and a catalytically inactive mutant of E6AP, C833A, were purchased from Addgene (Cambridge, MA, USA). Transfection of LX-2 cells were done with pCMV4 (MOCK), pCMV4-HA-E6AP (E6AP), or pCMV4-HA-E6AP C833A for 24 h using Lipofectamine^TM^2000 (Invitrogen). For the regulation of miRNA expressions, cells were transiently transfected with 100 nM each of control mimic, miR-302c mimic, miR-302c inhibitor, or respective negative control miRNA, using Lipofectamine^TM^2000 reagent according to the manufacturer’s protocol; miRNA mimic control, has-miR-302c mimic, inhibitor control, and inhibitor has-miR-302c were obtained from Bioneer. For gene knockdown, transfection of cells were performed with control siRNA (100 pmol) or E6AP siRNA (100 pmol) (Ambion, Austin, TX, USA) for 24 h using Lipofectamine^TM^2000. The cells were transfected with a plasmid using Lipofectamine^TM^2000 in Eagle’s minimum essential medium (MEM) for 3 h. The medium was then changed to MEM containing 1% FBS.

### Luciferase assays

Cells were transiently transfected with pRL-TK plasmid (a plasmid that encodes Renilla luciferase for normalization of transfection efficacy), and SBE or AP-1 luciferase plasmid using Lipofectamine^TM^2000 (Invitrogen) for 3 h. Transfected cells were recovered in MEM containing 1% FBS overnight and then incubated to TGF-β for 3 h. The firefly and Renilla luciferase activities were measured using the dual luciferase assay system (Promega, Madison, WI, USA). Relative luciferase activities were calculated by normalization of firefly luciferase activities based on those of Renilla luciferase. For E6AP 3′-UTR luciferase activities, the plasmid containing the Luc-E6AP-3′-UTR construct (Product ID: HmiT018388-MT06) used in reporter assays was purchased from GeneCopoeia (Rockville, MD, USA). The plasmid contains firefly luciferase fused to the 3′-UTR of human E6AP and Renilla luciferase that functions as a tracking gene. Luciferase activity assays were performed according to the manufacturer’s protocol. The luciferase activities were measured sequentially with the dual luciferase assay kit (GeneCopoeia). The activities were normalized with Renilla luciferase activities and expressed in relative luciferase activity units.

### Confocal microscopy

Briefly, primary HSCs were grown on a coverslip and fixed in a 4% paraformaldehyde solution, followed by permeabilization with 0.1% Triton X-100. The cell samples were immunostained with antibodies directed against E6AP overnight, followed by incubation with Alexa Fluor^®^ 488 goat anti-rabbit IgG (Invitrogen). Tissue sections were deparaffinized and incubated with antibodies of E6AP and desmin overnight and 4 h at 37 °C, respectively, followed by incubation with Alexa Fluor^®^ 594 goat anti-rabbit IgG (Biolegend, San Diego, CA, USA) or Alexa Fluor^®^ 488 goat anti-rabbit IgG (Biolegend) at 37 °C for 3 h. After incubation, the samples were cover-slipped with mounting media. The samples were examined using a laser-scanning confocal microscope (A1, Nikon instruments Inc., NY, USA).

### Statistical analysis

To assess significant differences among treatment groups, One-way ANOVA was used. For each significant treatment effect, the Newman-Keuls test was adopted to compare multiple groups. Results are expressed as mean ± standard error (SE).

## Supplementary information

Supplementary file.

## Data Availability

The datasets generated during and/or analyzed during the current study are available from the corresponding author on reasonable request.
