# Supplementary material for: Induction of E6AP by microRNA-302c dysregulation inhibits TGF-β-dependent fibrogenesis in hepatic stellate cells
Source: Sci Rep. 2020 Jan 16;10:444. doi: 10.1038/s41598-019-57322-w (PMC6965100; doi:10.1038/s41598-019-57322-w)
Supplement: Supplementary file 1 — Supplementary file. [file 41598_2019_57322_MOESM1_ESM.pdf]

Induction of E6AP by microRNA-302c dysregulation inhibits TGF-β-dependent fibrogenesis in hepatic stellate cells

Ji Young Kim[1,†], Kyu Min Kim[1,†], Ji Hye Yang[1,2], Sam Seok Cho[1],
Seung Jung Kim[1], Su Jung Park[1], Sang-Gun Ahn[3], Gum Hwa Lee[1],
Jin Won Yang[4], Sung Chul Lim[5], Keon Wook Kang[6], Sung Hwan Ki[1*]

[1]College of Pharmacy, Chosun University, Gwangju 61452, Republic of Korea.
[2]College of Korean Medicine, Dongshin University, Naju, Jeollanam-do 58245, Republic of Korea
[3]Department of Pathology, College of Dentistry, Chosun University, Gwangju 61452, Republic of Korea.
[4]College of Pharmacy, Woosuk University, Wanju, Jeonbuk, 55338, Republic of Korea.
[5]College of Medicine, Chosun University, Gwangju 61452, Republic of Korea.
[6]College of Pharmacy and Research Institute of Pharmaceutical Sciences, Seoul National University, Seoul 08826, Republic of Korea.

Raw Data Figures

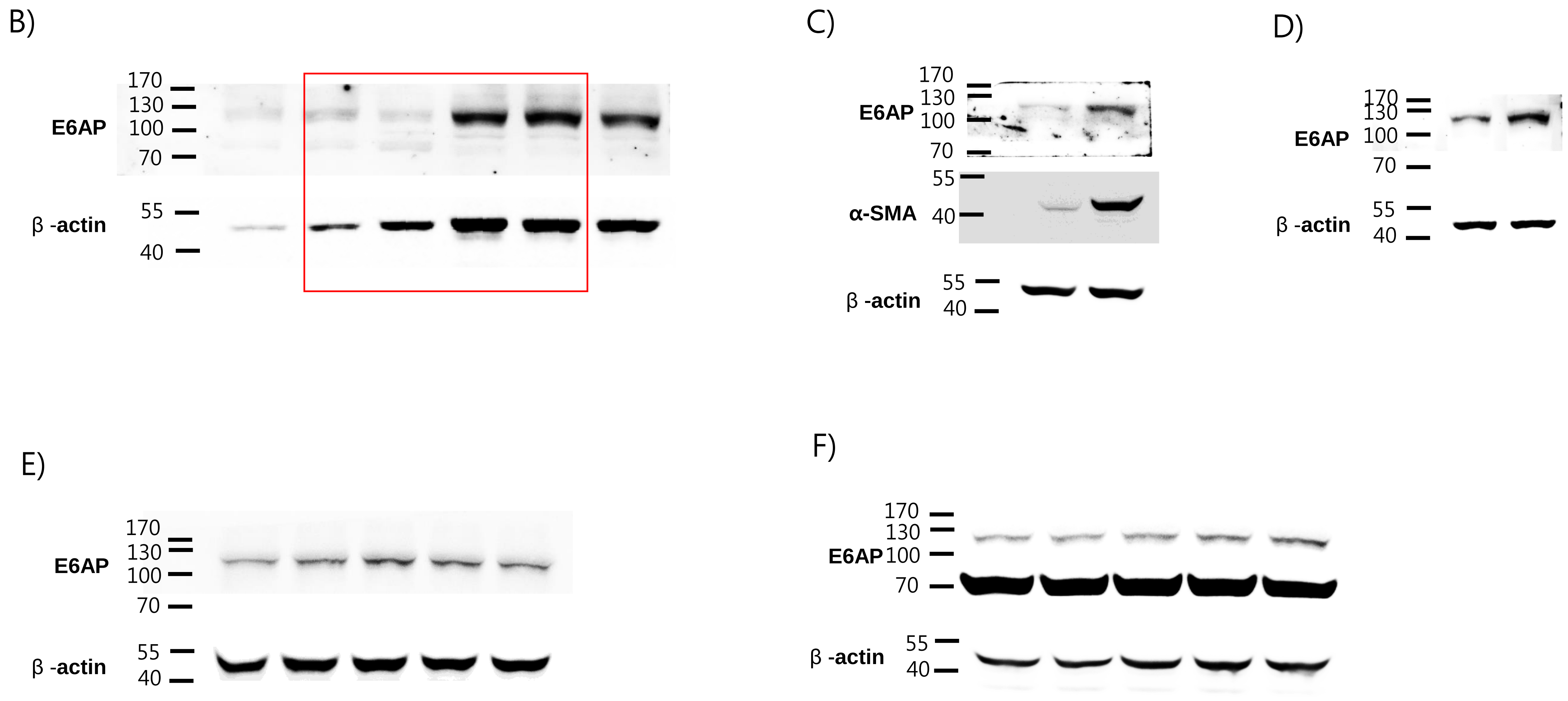

Supplementary Fig 1. Raw data of Figure 1

C)

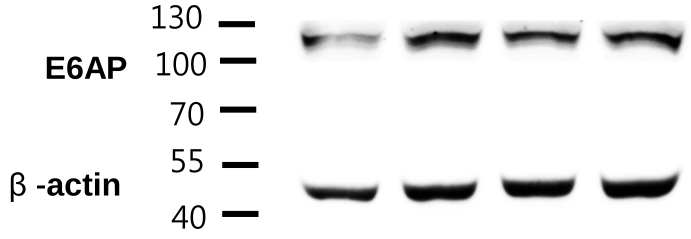

D)

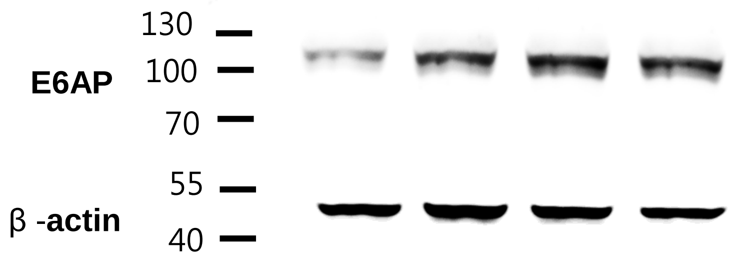

E)

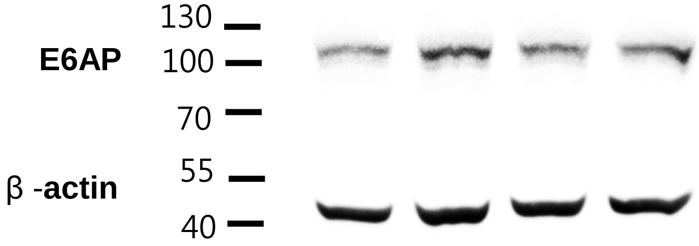

Supplementary Fig 2. Raw data of Figure 2

D)

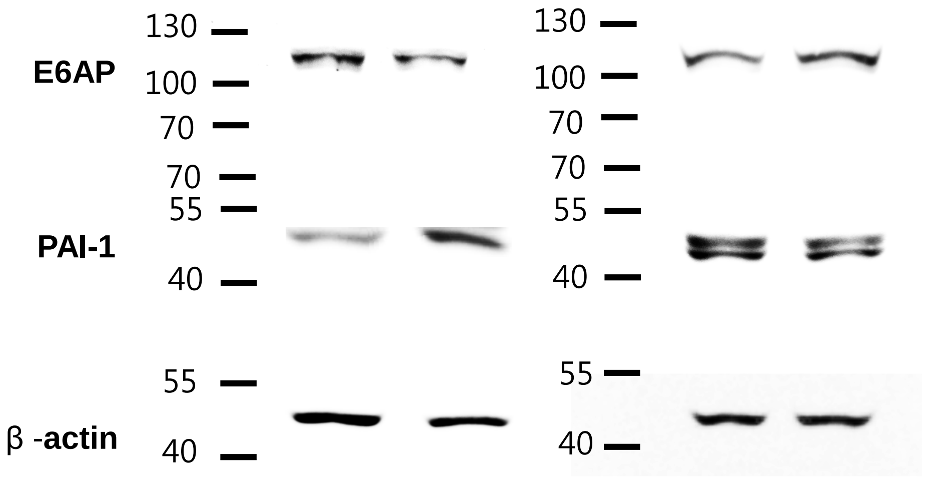

Supplementary Fig 3. Raw data of Figure 4

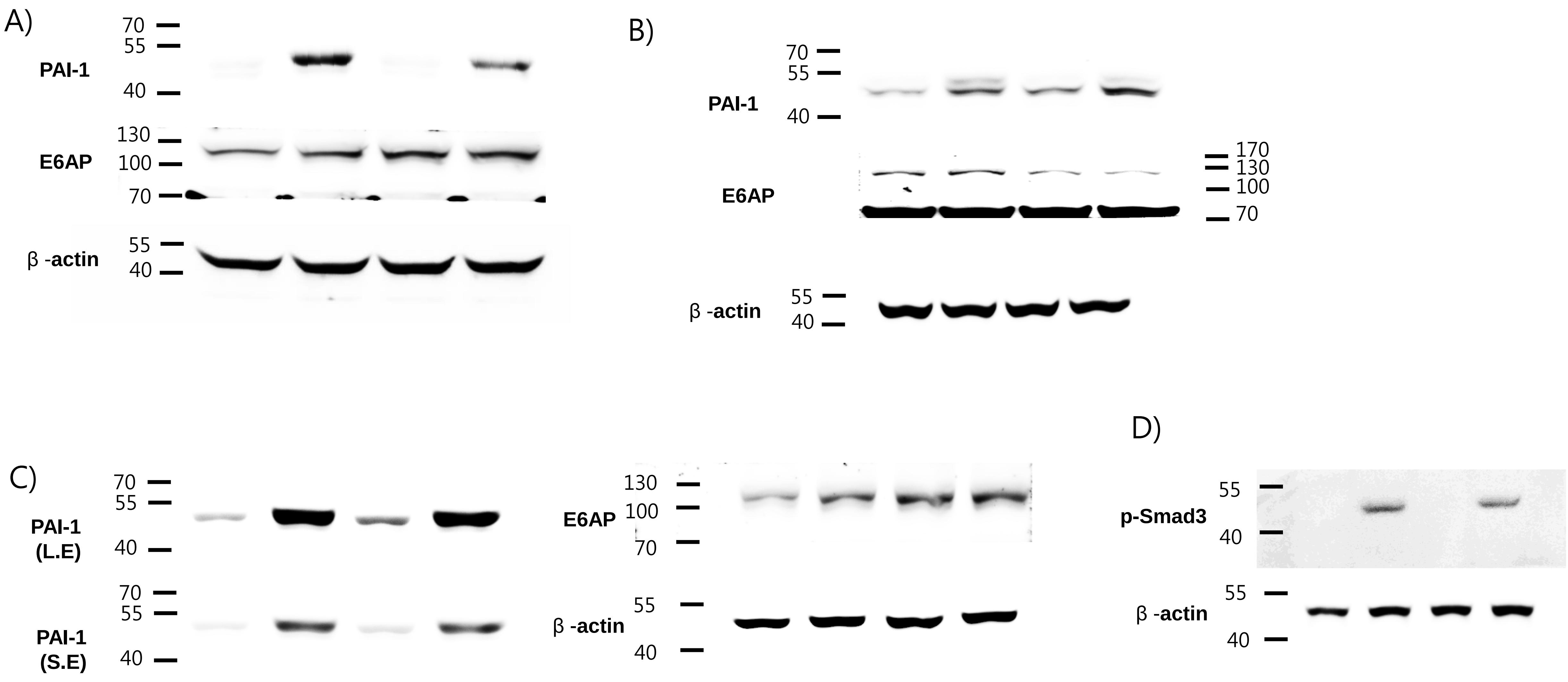

Supplementary Fig 4. Raw data of Figure 5

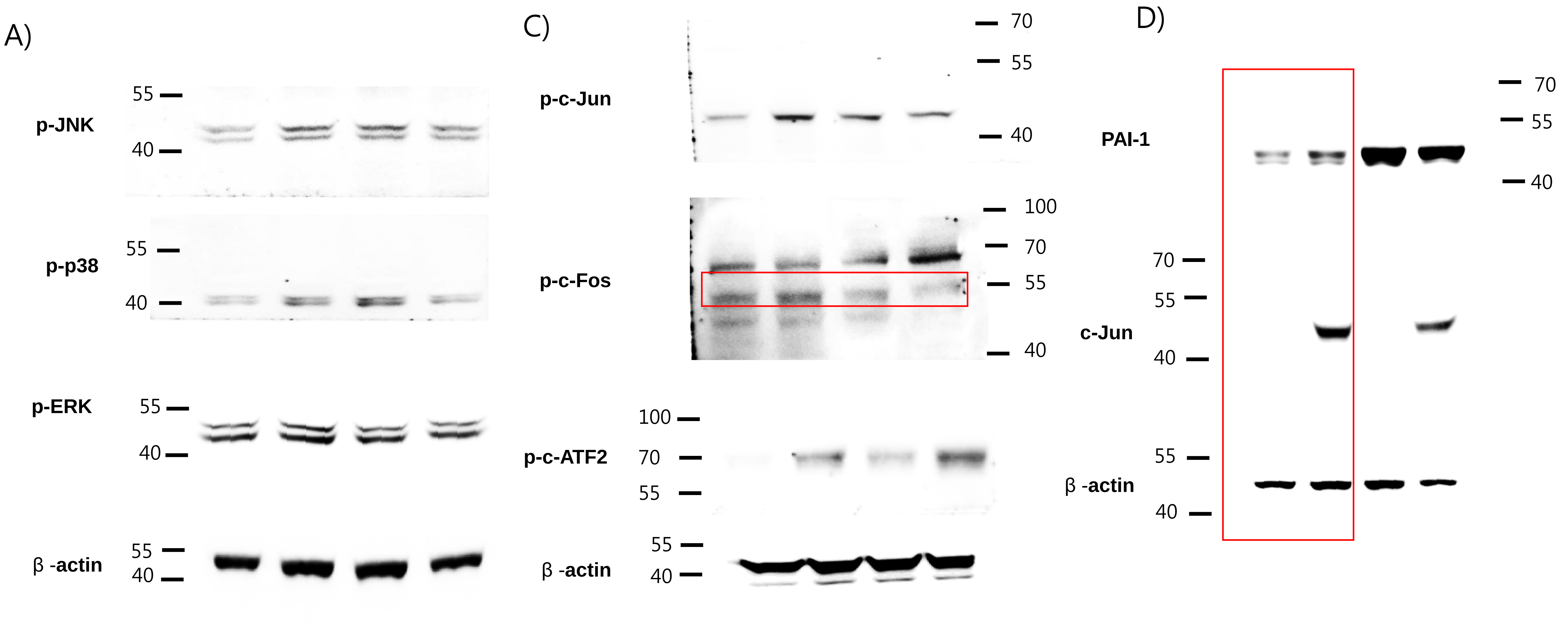

Supplementary Fig 5. Raw data of Figure 6
